# Pollution and Health Risk Assessments of Potentially Toxic Elements in the Fine-Grained Particles (10–63 µm and <10 µm) in Road Dust from Apia City, Samoa

**DOI:** 10.3390/toxics10110683

**Published:** 2022-11-11

**Authors:** Hyeryeong Jeong, Kongtae Ra

**Affiliations:** 1 Ifremer, Département Ressources Biologiques et Environnement (RBE), Unité Contamination Chimique des Ecosystèmes Marins (CCEM), F-44000 Nantes, France; 2 Marine Environmental Research Center, Korea Institute of Ocean Science and Technology (KIOST), Busan 49111, Korea; 3 Department of Ocean Science (Oceanography), KIOST School, University of Science and Technology (UST), Daejeon 34113, Korea

**Keywords:** potentially toxic elements, source identification, risk assessment, Samoa

## Abstract

Fine road dust is a major source of potentially toxic elements (PTEs) pollution in urban environments, which adversely affects the atmospheric environment and public health. Two different sizes (10–63 and <10 μm) were separated from road dust collected from Apia City, Samoa, and 10 PTEs were analyzed using inductively coupled plasma mass spectrometry (ICP-MS). Fine road dust (<10 μm) had 1.2–2.3 times higher levels of copper (Cu), zinc (Zn), arsenic (As), cadmium (Cd), antimony (Sb), lead (Pb), and mercury (Hg) than 10–63 μm particles. The enrichment factor (EF) value of Sb was the highest among PTEs, and reflected significant contamination. Cu, Zn, and Pb in road dust were also present at moderate to significant levels. Chromium (Cr), cobalt (Co), and nickel (Ni) in road dust were mainly of natural origins, while Cu, Zn, Sb, and Pb were due to traffic activity. The levels of PTEs in road dust in Samoa are lower than in highly urbanized cities, and the exposure of residents in Samoa to PTEs in road dust does not pose a noncarcinogenic health risk. Further studies of the effects of PTEs contamination in road dust on the atmosphere and living organisms are needed.

## 1. Introduction

There have been many studies on metal contamination in road dust in association with increasing transportation activities worldwide, due to urbanization and industrialization [1,2,3,4,5]. In urban environments, road dust is highly contaminated with various potentially toxic elements (PTEs) of natural origin, as well as PTEs derived from human activities, including industrial and traffic emissions [6,7]. These PTEs do not decompose in the environment and accumulate in living organisms. Some metals in road dust, including chromium (Cr), copper (Cu), zinc (Zn), antimony (Sb), cadmium (Cd), and lead (Pb), are derived from the corrosion of plated parts of vehicles and wear of brake pads, discs, tires, and asphalt [8,9,10,11]. Although some metals, such as Cu and Zn, are classified as essential, high concentrations in the environment have toxic effects on living organisms [12,13]. Some metals (Cr, Cd, and Pb) are classified as toxic elements and can present a carcinogenic risk to humans [14,15,16].

Fine road dust particles generally have relatively high PTEs concentrations compared to coarse particles due to their large surface area, and PTEs concentrations tend to increase as the particle size becomes smaller [17,18,19]. Fine particles of road dust are resuspended by wind and rainfall runoff, transported into the atmosphere and surrounding aquatic environments, and eventually deposited in coastal areas [20,21,22,23,24]. Therefore, road dust is a major source of PTEs contamination in urban environments, as well as the atmosphere and coastal environments. PTEs are of interest due to concerns about the detrimental effects of contaminated road dust on the atmosphere, living organisms, environment, and human health. Many countries are working to reduce the harmful effects of road and street dust on the environment and public health by regularly cleaning road surfaces using sweeping machines and vehicles to manage atmospheric pollution and stormwater quality [25,26,27,28,29]. These efforts have reduced the amount of road dust and metal contamination, but are limited to urban areas with large traffic volumes and high population density.

The fine particles in road dust are not removed efficiently, so they accumulate on the road surface over long periods. PTEs concentrations in road dust have been reported to be relatively high in areas of heavy traffic, including industrial, residential, and commercial areas [30,31,32]. Relatively high concentrations of PTEs were found in river sediments near urban and commercial regions, and PTEs-contaminated road dust is considered as a cause of contamination to river and coastal environments [24,33]. As more than half of the world’s population lives in urban cities, residents living in areas with heavy traffic and high population densities are at higher risk of exposure to PTEs present in road dust [34,35]. Especially, road dust with a diameter <10 μm can enter the human body through inhalation, ingestion, and dermal contact [36,37,38]. Long-term exposure adversely affects human health, and may even have carcinogenic effects.

Studies on PTEs in road dust are concentrated in large cities and industrial regions, and the human health risk of residents and employees are evaluated. In Samoa, the tourism industry is making an important economic contribution. Traffic activities related to tourism is one major cause of PTE contamination in road dust and can have a detrimental effect on residents as well as tourists. However, research on PTE contamination and risk assessments in road dust from Pacific Island countries, including Samoa, is very limited. Therefore, this study was performed to evaluate PTE contamination and health risks posed by fine road dust (10–63 μm, <10 μm) collected in Apia City, Samoa.

## 2. Materials and Methods

### 2.1. Study Area

Samoa is located in the Polynesian region of south-central Pacific Ocean and consists of the two large island of Upolo and Savai’i and eight small islands. Our study area, Upolo island, is a volcanic island formed by a massive basaltic shield volcano rising from the seafloor of the Pacific Ocean. The volcanic rocks of Upolo Island include basaltic lavas, scoria cones, tuffs, and pyroclastic [39,40]. There is mountainous interior covered with dense rain forests with a height about 1100 m of volcanic origin. The length and area of Upolo Island are 75 km and 1125 km^2^, respectively, and Apia, the capital of Samoa, is located in the northern part of the Upolu Island. The climate of study area is oceanic tropical characterized by high humidity and temperature.

### 2.2. Sampling and PTE Analysis

**Figure 1:**
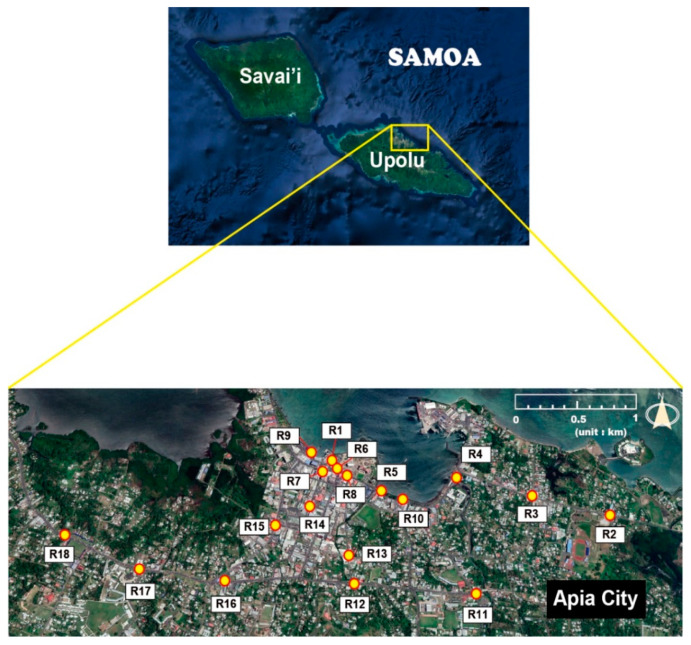
Map of sampling sites for road dust from Apia city of Samoa (base map from Google Earth).

Eighteen road dust samples were collected using a vacuum cleaner (DC35; Dyson, Wiltshire, UK) from Apia City, Samoa, in July 2014 (Figure 1). To prevent cross-contamination during road dust sampling, the vacuum cleaner was disassembled and cleaned after sampling.

Four surface soil samples were also collected near the R2, R11, R12, and R16 sites to provide background values for pollution assessment. In the laboratory, particles of two size classes (10–63 and <10 μm) were separated using a vibratory sieve shaker (Analysette 3 Pro; Fritsch Co., Idar-Oberstein, Germany) with a nylon sieve. For PTE analysis, all samples were ground and homogenized with a planetary mono mill (Pulverisette 6; Fritsch Co.).

After weighing about 100 mg of sample in a Teflon vessel, high-purity acid mixture (HF, HNO_3_, HClO_4_ = 4:3:1 *v*/*v*) was added and heated at 180 °C for 24 h on a hot plate for complete digestion. The decomposed samples were evaporated to dryness and dissolved in 2% HNO_3_. PTEs other than Hg were analyzed by inductively coupled plasma mass spectrometry (ICP-MS; i-CAPQ; Thermo Fisher Scientific, Dreieich, Germany) at the Korea Institute of Ocean Science and Technology (KIOST). Hg concentrations were determined using a direct mercury analyzer (DMA-80; Milestone Inc., Sorisole, Italy).

Two certified reference materials (CRMs), MESS4 and BCR723, were decomposed together with the samples and analyzed to verify the reliability of PTE analysis. The recovery of CRMs was 97.6–103.3% for MESS-4 (*n* = 6) and 96.2–106.1% for BCR723 (*n* = 6), which were highly consistent with the certified values.

### 2.3. Pollution Assessment

Many pollution indices are widely used to evaluate the contamination levels of single or multiple elements. In this study, the enrichment factor (EF) and pollution load index (PLI) were used to evaluate PTE contamination in road dust.

EF was calculated by the following equation, and aluminum (Al) was used as a reference element.

$$EF|=|\frac{{\left(metal/Al\right)}_{sample}}{{\left(metal/Al\right)}_{background}}$$

where (metal/Al)_sample_ and (metal/Al)_background_ are the ratios of PTE and Al concentrations in two different size classes of road dust and background soil, respectively. The concentrations of Cr, Co, Ni, Cu, Zn, As, Cd, Sb, Pb, and Hg in surface soil, as background values, were 519, 96.3, 346, 37.9, 109, 7.8, 0.33, 0.50, 13.1, and 0.05 mg/kg, respectively (Table 1).

The calculated EF values were classified into five categories as follows [41]: EF < 2 (depletion to minimum enrichment), 2 < EF < 5 (moderate enrichment), 5 < EF < 20 (significant enrichment), 20 < EF < 40 (very high enrichment), EF > 40 (extremely high enrichment).

PLI was evaluated using the following equation, reflecting the overall contamination level of 10 PTEs in road dust analyzed in this study [42].

PLI∣=∣(Ci1Cb1∣×∣Ci2Cb2∣×∣Ci3Cb3∣×∣⋯CinCbn)1n

where C_i_ and C_b_ are the concentrations of element *i* in road dust and background soil, respectively.

The calculated PLI values were divided into four categories, as follows [43,44]: PLI < 1 (unpolluted), 1 < PLI < 2 (low polluted), 2 < PLI < 3 (moderately polluted), and PLI > 3 (heavily polluted).

### 2.4. Health Risk Assessment

The noncarcinogenic health risks to adults and children were evaluated considering three exposure pathways, i.e., ingestion (hand to mouth), inhalation (mouth and nose), and dermal contact. The level of PTE exposure by fine road dust particles (<10 μm) was calculated by the following equation using the chronic daily intake [45].

$${\mathrm{ADD}}_{\mathrm{ing}}|=|C_{i}|\times |\left(\frac{\mathrm{IngR}|\times |\mathrm{EF}|\times |\mathrm{ED}}{\mathrm{BW}|\times |\mathrm{AT}}\right)|\times |10^{-6}$$

$${\mathrm{ADD}}_{\mathrm{inh}}|=|C_{i}|\times |\left(\frac{\mathrm{InhR}|\times |\mathrm{EF}|\times |\mathrm{ED}}{\mathrm{PEF}|\times |\mathrm{BW}|\times |\mathrm{AT}}\right)$$

$${\mathrm{ADD}}_{\mathrm{derm}}|=|C_{i}|\times |\left(\frac{\mathrm{SL}|\times |\mathrm{SA}|\times |\mathrm{ABS}|\times |\mathrm{EF}|\times |\mathrm{ED}}{\mathrm{BW}|\times |\mathrm{AT}}\right)|\times |10^{-6}$$

Here, C_i_ is the concentration of PTE i in road dust (<10 μm).

The noncarcinogenic health risk for each PTE was calculated using the following equation.

$${\mathrm{HQ}}_{\mathrm{ing}}|=|\frac{{\mathrm{ADD}}_{\mathrm{ing}}}{{\mathrm{RfD}}_{\mathrm{ing}}};\;{\mathrm{HQ}}_{\mathrm{inh}}|=|\frac{{\mathrm{ADD}}_{\mathrm{inh}}}{{\mathrm{RfD}}_{\mathrm{inh}}};\;{\mathrm{HQ}}_{\mathrm{derm}}|=|\frac{{\mathrm{ADD}}_{\mathrm{derm}}}{{\mathrm{RfD}}_{\mathrm{derm}}}$$

The hazard index (HI) was calculated as the sum of the HQ values for the three exposure pathways.

HI values > 1 indicate potential chronic effects, and HI < 1 indicates no risk of adverse health effects [46].

RfD is the reference dose for ingestion, inhalation, and dermal contact, and is an estimate of daily exposure in residents [47,48]. The values for each parameter in the ADD calculation were obtained from Adamiec and Jarosz-Krzeminska [49] and are presented by Jeong et al. [4].

### 2.5. Statistical Analysis

The mean, minimum, maximum, and coefficient of variation (CV) values were carried out using Microsoft Excel program based on the PTE concentration. The Pearson’s correlation analysis was applied to understand the potential source of each PTE in road dust (<10 μm) through the correlation between elements and was performed using PASW Statistics 18.0.

## 3. Results and Discussion

### 3.1. PTE Concentration

**Figure 2:**
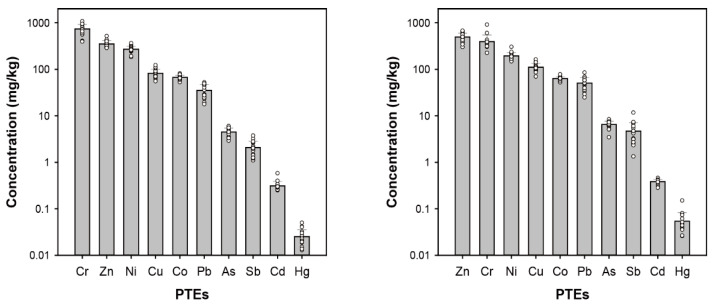
Comparison of PTEs concentrations with particle size of 10–63 μm (**left**) and <10 μm (**right**) in road dust.

The minimum, maximum, mean, and coefficient of variation (CV) PTE levels in road dust are presented in Table 1. Figure 2 shows 10 PTE concentrations for particle sizes in road dust of 10–63 and <10 μm. In road dust with particle size 10–63 μm, Cr had the highest mean concentration and the PTE concentrations decreased in the order Cr (739 mg/kg) > Zn (351 mg/kg) > Ni (272 mg/kg) > Cu (82.8 mg/kg) > Co (66.7 mg/kg) > Pb (35.3 mg/kg) > As (4.5 mg/kg) > Sb (2.1 mg/kg) > Cd (0.31 mg/kg) > Hg (0.03 mg/kg) (Table 1) [47,48,49]. The CV represents the spatial variation according to sampling site for the PTE concentrations analyzed in this study. A high CV reflects heterogeneity of the PTE concentration among environmental samples, indicating the presence of hotspots of PTE contamination from specific anthropogenic sources [50,51]. The CVs for Cr, Ni, and Zn were <20%, indicating low regional variability, while the other PTEs showed moderate regional variability (20% < CV < 50%).

In road dust with particle size <10 μm, Zn showed the highest mean concentration, at 494 mg/kg (Figure 2). The mean concentrations of Cr, Co, and Ni in road dust with particle size 10–63 μm were higher than those in fine road dust (<10 μm). However, the fine road dust had 1.2–2.3 times higher levels of Cu, Zn, As, Cd, Sb, Pb, and Hg than road dust with particle size 10–63 μm.

**Table 1:** Comparison of mean values (minimum and maximum value in parenthesis) and coefficient of variation (CV; %) for Al and PTEs concentration in the fine particles of road dust from Samoa and those in the other reported data (mean value).

Size		Al	Cr	Co	Ni	Cu	Zn	As	Cd	Sb	Pb	Hg	References
		%	mg/kg	mg/kg	mg/kg	mg/kg	mg/kg	mg/kg	mg/kg	mg/kg	mg/kg	mg/kg	
10–63 μm	mean min. max. CV (%)	5.8 4.9 6.6 8	739 395 1078 26	66.7 53.0 81.6 11	272 184 364 16	82.8 55.5 123 21	351 288 520 18	4.49 2.92 5.99 21	0.31 0.25 0.58 25	2.08 1.10 3.76 36	35.3 17.7 52.6 33	0.03 0.01 0.05 40	This study
<10 μm	mean min. max. CV (%)	7.0 5.8 8.5 9	395 226 913 38	62.9 52.3 78.0 9	195 149 304 18	111 70.0 163 21	494 301 677 21	6.5 3.5 8.4 18	0.38 0.28 0.46 12	4.68 1.34 11.7 53	50.5 24.8 85.0 31	0.05 0.03 0.15 18	This study
Soil	mean	8.5	519	96.3	346	37.9	109	7.8	0.33	0.50	13.1	0.05	This study
<10 μm	mean	-	300	19.8	75.6	513	3007	17.0	5.6	61.6	480	0.6	Korea [52]
<10 μm	mean	-	53.8	9.3	34.2	100	302	17.0	0.6	-	48.8	-	China [53]
<10 μm	mean	-	68	15	46	184	1026	-	0.8	12	91	-	Russia [54]

The highest concentrations of Cu (163 mg/kg), Zn (677 mg/kg), and Sb (11.69 mg/kg) were observed at site R17, which had a high traffic volume. Pb (85.0 mg/kg) and Hg (0.15 mg/kg) concentrations were highest at site R5, which is near the intersection in the downtown direction of Apia City. PTE concentrations tended to be relatively high at sampling sites with high traffic volume. The CV of Sb was 53%, which was higher than those of other PTEs. Most PTEs showed low CVs and the spatial distributions in fine road dust were not different. These results suggest that PTEs in road dust may be affected by human activities, such as transportation of residents and tourists, rather than by specific contamination sources, such as industrial emissions.

### 3.2. Pollution Assessment of PTEs in Road Dust

**Figure 3:**
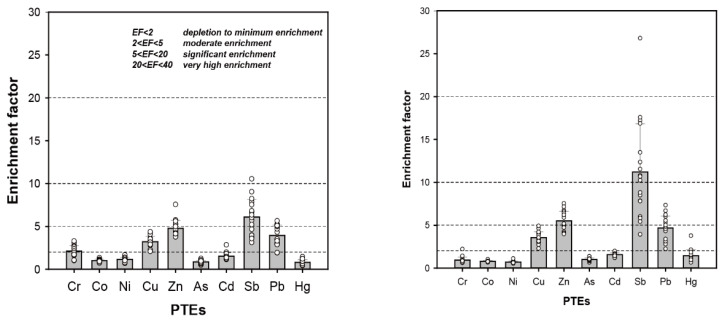
Comparison of enrichment factor (EF) for PTEs with particle size 10–63 μm (**left**) and <10 μm (**right**) in road dust.

The mean EF values for 10 PTEs are shown in Figure 3. Sb had the highest mean EF value among the PTEs examined in both fractions of road dust. The mean EF values of PTEs in road dust with a particle size of 10–63 μm decreased in the order Sb > Zn > Pb > Cu > Cr > Cd > Ni > Co > As > Hg, whereas those in fine road dust (<10 μm) decreased in the order Sb > Zn > Pb > Cu > Cd > Hg > As > Cr > Co > Ni; the EF values for Cr, Co, and Ni, which are of natural origin, were relatively low. For 10–63-μm road dust particles, the mean EF value of Sb was 6.1, corresponding to significant contamination. Cu, Zn, and Pb had mean EF values between 2 and 5, indicating moderate contamination. For Zn, the EF values exceeded 5 at five sampling sites with high traffic volume.

For fine road dust (<10 μm), the mean EF of Sb was 11.2, corresponding to significant contamination. At sampling site R17, which had the widest road and dense traffic, the EF value of Sb was 26.8, indicating very high contamination. The mean EF of Zn, contamination with which is mainly due to tire wear in urban areas, indicates significant contamination. The contamination level of road dust in Samoa was lower than in large cities with high population density and traffic activity, but high levels of Cu, Zn, Sb, and Pb contamination were observed in relation to traffic activity (Table 1). Notably, Sb, which is present in high levels in brake pads [9,55], was a significant contaminant.

**Figure 4:**
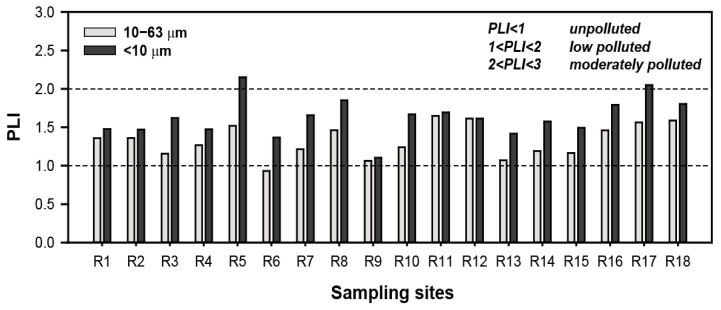
Spatial distribution of pollution load index (PLI) in road dust at sampling site.

The PLI value of road dust with particle size of 10–63 μm ranged from 0.9 to 1.6 and (mean value = 1.3), indicating low pollution levels for 10 PTEs (Figure 4). The mean PLI value of fine road dust (<10 μm) was 1.6, corresponding to low pollution. However, two sampling sites (R5 and R17) had higher PLI values (exceeding 2) than the other sampling sites 2, indicating that that fine road dust in Samoa was moderately polluted with PTEs.

Table 2 shows the relationship between the concentration of PTEs in <10 μm of road dust of this study. The concentrations of Cr, Ni, and Co in road dust were closely related. The mean concentrations of Cr, Ni, and Co in the surface soil were 519, 96.3, and 346 mg/kg, respectively, which were very high compared to road dust in this study and the upper continental crust [56]. In a study of heavy metals in soils of a volcanic island (Réunion) located in the western part of the Indian Ocean, Dœlsch et al. [57] reported that the Cr and Ni contents in soil were closely related to volcanic rocks. In this study, no clear correlations were found between Cr, Ni, or Co, and other PTEs (Cu, Zn, Sn, and Pb), mainly arising from nonexhaust emission sources, such as the wear of tires, brake pads, and asphalt due to traffic activity. However, there were significant correlations among Cu, Zn, Sb, and Pb. Therefore, the high Cr, Ni, and Co concentrations in road dust in Samoa, and their high correlations, were strongly influenced by natural processes, such as the weathering of volcanic parent rocks.

### 3.3. Non-Carcinogenic Risk of PTEs in Fine Road Dust (10 μm)

Table 3 presents the mean HQ and HI values for adults and children, and three exposure pathways (in descending order): HQ_ing_ > HQ_derm_ > HQ_inh_. These observations indicated that the pathway of ingestion from hand to mouth was the main exposure route, having a detrimental effect on human health compared to other processes. The contribution of ingestion was 76% for adults and 79% for children, but the sum of the HQ values for PTEs was 0.62 for children, which was higher than that for adults (0.07). HQ and HI values for all PTEs were <1 at all sampling sites, indicating no noncarcinogenic health risk for adults or children. The mean HI values for adults and children were 0.09 and 0.78, respectively. Therefore, the noncarcinogenic health risk of exposure to PTEs in road dust was about nine times higher in children than adults.

The HI value of Cr was higher than that of other PTEs, and the order of HI values was Cr > As > Sb > Pb > Ni > Co > Cu > Zn > Cd > Hg in adults. These results suggest that Cr and As may be major contributors to noncarcinogenic risk among PTEs in road dust accumulated on road surfaces in Samoa.

Cr is widely used in many industries, including metallurgy, steel manufacturing, automobile industry, electroplating, and wood preservation, because of its anticorrosive properties [58,59,60,61]. Cr is highly mobile and has extremely toxic effects even at very low concentrations; it is classified as a Group A carcinogen element in humans [62]. Cr is used for the wheels of vehicles, and many parts of vehicles are plated with Cr as it has an aesthetic appearance and is not easily corroded. Many studies have reported that Cr contamination in road dust and soil is due to traffic and industrial activities [63,64,65,66].

Although the noncarcinogenic risk of Cr is higher than those of other PTEs, the mean EF value of Cr in road dust (<10 μm) was 0.9 (range 0.6–2.2) in this study, and most of the sampling sites showed depletion to minimal enrichment. The contamination level of Cr in road dust was lower than those of Cu, Zn, Sb, and Pb, which are mainly derived from traffic activity. High concentrations of Cr and Ni have been reported in the soil of islands formed by volcanic activity [57,67].

The mean concentrations of Cr in road dust (<10 μm) and surface soil were 395 and 519 mg/kg, respectively, in this study, which were higher than those in the upper continental crust (92.0 mg/kg) [55]. Our results indicated that Cr concentration in road dust in Samoa is derived from natural sources, mainly due to the weathering of volcanic parent materials. Therefore, the noncarcinogenic risk of Cr in road dust on Samoa seemed to be much lower than in highly urbanized cities.

PTEs such as Cu, Zn, Sb, and Pb have a negligible health risk on the human body, but road dust is contaminated with these elements which come from traffic activities. Pacific Island countries, including Samoa, import and use used cars for the tourisms industry. The activation of the tourism industry and the environmental factors (high humidity and heavy precipitation) can increase the contamination levels of PTEs in road dust, as these accelerate the corrosion of vehicles. In addition, road dust contaminated with PTEs is easily resuspended via stormwater runoff, transport to rivers and streams, and is finally deposited in the coastal environments. Road dust in urban environments is one of the major sources of PTEs contamination that contaminates marine sediments and has a detrimental effect on marine organisms. Efficient management by road cleaning is necessary to prevent the impact on the environments and ecosystem caused by road dust contaminated with PTEs.

## 4. Conclusions

In this study, road dust was collected in Apia City, Samoa, and divided into two fractions (10–63 and <10 μm). Analyzing road dust is important for public health, and for understanding environmental PTE contamination. Pollution and health risk assessments were performed for 10 PTEs in road dust. Cr, Co, and Ni concentrations in road dust with a particle size of 10–63 μm were higher than in fine road dust (<10 μm), while Cu, Zn, As, Cd, Sb, Pb, and Hg were present at relatively high concentrations in fine road dust. The PTE concentration in road dust was lower than in large cities with high levels of human activity. The CVs of PTEs in road dust suggested that anthropogenic sources related to traffic activity rather than industrial emissions affect road dust in Apia City, Samoa. Both particle sizes of road dust had significant levels of Sb, and the levels of Cu, Zn, and Pb, which are derived from nonexhaust emission arising from traffic activity, were also moderately high. The results of correlation analyses and pollution assessments showed that Cr, Co, and Ni are greatly affected by natural sources due to the weathering of volcanic parent rocks. There were significant correlations among Cu, Zn, Sb, and Pb. In addition, relatively high concentrations were observed in sampling sites with high traffic volume, suggesting that contamination with these PTEs (Cu, Zn, Sb, and Pb) was mainly due to traffic activity. The ingestion exposure route posed a greater health risk than inhalation and dermal contact. The mean HI values for adults and children were 0.09 and 0.78, respectively, indicating that the noncarcinogenic health risk in children was about nine times higher than that in adults. However, HI values for adults and children were <1 at all sampling sites, suggesting no noncarcinogenic risk from exposure to PTEs in road dust. Among the PTEs, Cr had a higher HQ value than the other elements, and posed a major noncarcinogenic health risk. The Cr in road dust in Samoa was mainly derived from natural sources, i.e., the weathering of volcanic parent rocks rather than anthropogenic sources (traffic activity). Therefore, our results suggest that the health risks posed by PTEs in road dust in Samoa are negligible compared to highly urbanized cities. However, further studies regarding the effects of PTE-contaminated road dust on the atmosphere and living organisms, including humans, are needed.

## Figures and Tables

**Table 2:** Results of Pearson’s correlation matrix between PTEs in <10 μm of road dust. Bold indicates that correlation is significant at the 0.05 level (2-tailed).

	Cr	Ni	Co	Cu	Zn	As	Cd	Sb	Pb	Hg
Cr	1									
Ni	**0.72**	1								
Co	**0.95**	**0.82**	1							
Cu	−0.13	0.15	−0.13	1						
Zn	−0.36	−0.12	−0.40	**0.63**	1					
As	−0.26	−0.01	−0.22	0.29	0.45	1				
Cd	**−0.64**	−0.32	**−0.62**	0.47	**0.67**	**0.51**	1			
Sb	−0.16	−0.03	−0.17	**0.87**	**0.68**	0.26	**0.50**	1		
Pb	0.08	0.30	0.13	**0.63**	**0.57**	0.34	0.44	**0.51**	1	
Hg	0.11	0.30	0.23	0.22	0.25	0.30	0.10	0.16	**0.59**	1

**Table 3:** The median values of hazard quotient (HQ) and hazard index (HI) of non-carcinogenic hazards for PTEs with particle size of less than 10 μm in this study.

	Adult				Children			
	HQ_ing_	HQ_inh_	HQ_derm_	HI	HQ_ing_	HQ_inh_	HQ_derm_	HI
Cr	4.3 × 10^−2^	3.5 × 10^−3^	4.0 × 10^−2^	8.7 × 10^−2^	4.1 × 10^−1^	3.2 × 10^−2^	9.1 × 10^−2^	5.3 × 10^−1^
Co	1.1 × 10^−3^	3.0 × 10^−3^	3.2 × 10^−5^	4.1 × 10^−3^	1.0 × 10^−2^	2.8 × 10^−2^	7.3 × 10^−5^	3.8 × 10^−2^
Ni	3.3 × 10^−3^	2.5 × 10^−3^	2.8 × 10^−4^	3.6 × 10^−3^	3.1 × 10^−2^	2.4 × 10^−5^	6.4 × 10^−4^	3.2 × 10^−2^
Cu	9.5 × 10^−4^	7.3 × 10^−7^	7.3 × 10^−5^	1.0 × 10^−3^	8.9 × 10^−3^	6.8 × 10^−6^	1.7 × 10^−4^	9.1 × 10^−3^
Zn	5.9 × 10^−4^	4.5 × 10^−7^	6.7 × 10^−5^	6.6 × 10^−4^	5.5 × 10^−3^	4.2 × 10^−6^	1.5 × 10^−4^	5.7 × 10^−3^
As	7.7 × 10^−3^	5.9 × 10^−6^	4.3 × 10^−4^	8.2 × 10^−3^	7.2 × 10^−2^	5.5 × 10^−5^	9.9 × 10^−4^	7.3 × 10^−2^
Cd	1.4 × 10^−4^	1.0 × 10^−7^	3.1 × 10^−4^	4.5 × 10^−4^	1.3 × 10^−3^	9.7 × 10^−7^	7.2 × 10^−4^	2.0 × 10^−3^
Sb	3.8 × 10^−3^	2.9 × 10^−6^	4.4 × 10^−3^	8.2 × 10^−3^	3.6 × 10^−2^	2.7 × 10^−5^	1.0 × 10^−2^	4.6 × 10^−2^
Pb	5.0 × 10^−3^	3.8 × 10^−6^	7.7 × 10^−4^	5.8 × 10^−3^	4.7 × 10^−2^	3.6 × 10^−5^	1.8 × 10^−3^	4.9 × 10^−2^
Hg	5.4 × 10^−5^	4.1 × 10^−8^	1.7 × 10^−5^	7.1 × 10^−5^	5.0 × 10^−4^	3.8 × 10^−7^	4.0 × 10^−5^	5.4 × 10^−4^

## Data Availability

All data for this study are available from the first or corresponding authors upon request.
